# Supplementary material for: Saccharomyces boulardii (CNCM I-745) improves intestinal damage in sepsis by remodeling intestinal flora structure
Source: Front Cell Infect Microbiol. 2025 Jul 31;15:1602792. doi: 10.3389/fcimb.2025.1602792 (PMC12350329; doi:10.3389/fcimb.2025.1602792)
Supplement: Supplementary file 1 [file DataSheet1.zip › summary/summary/8_Advanced_analyse/2_PICRUSt2_result/KEGG/Group.BW4_vs_AW4/PICRUSt2_KEGG_level2_Group.BW4_vs_AW4_diff.pdf]

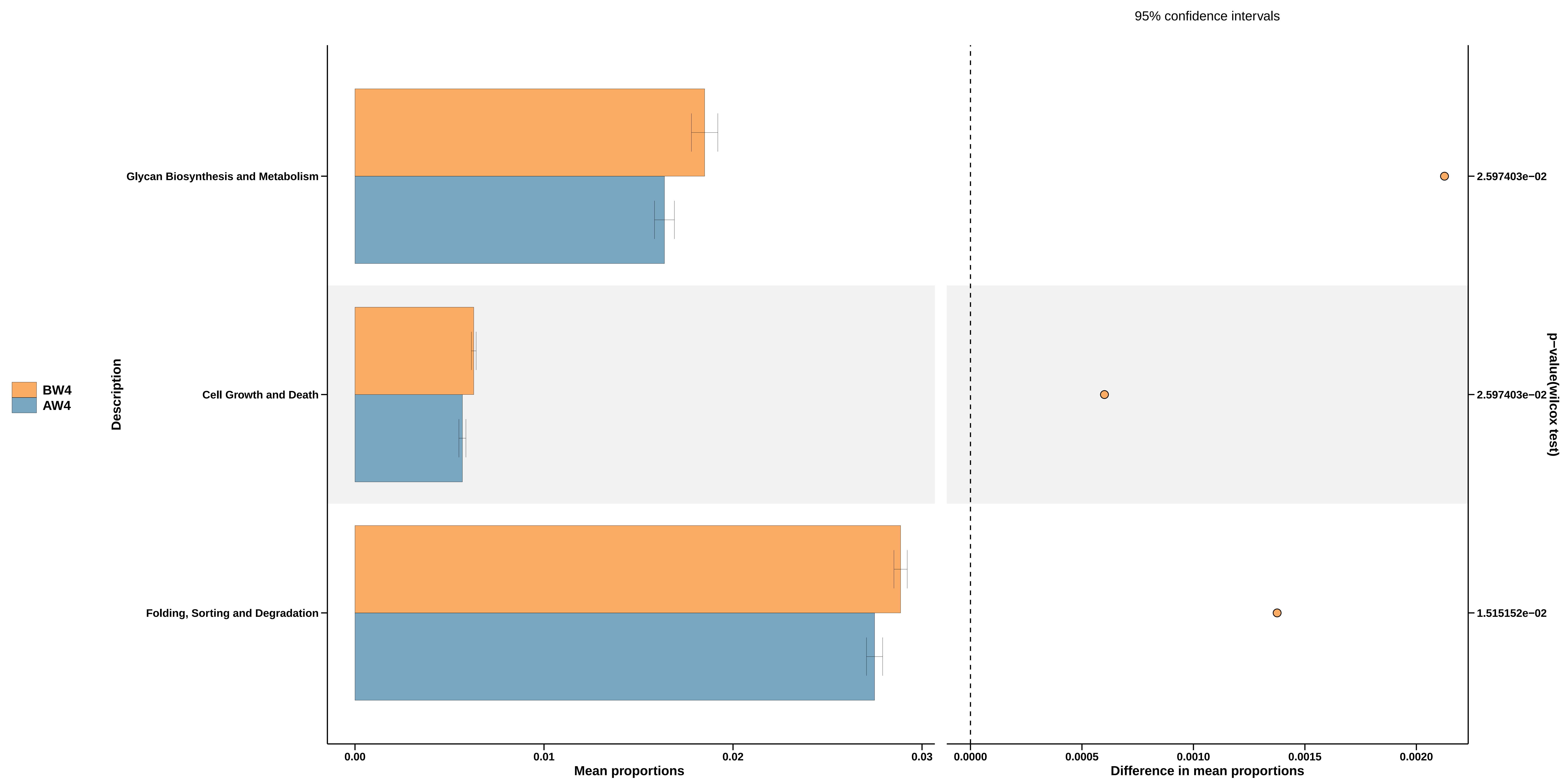

95% confidence intervals
BW4
AW4
Description
Glycan Biosynthesis and Metabolism
Cell Growth and Death
Folding, Sorting and Degradation
0.00
0.01
0.02
0.03
Mean proportions
0.0000
0.0005
0.0010
0.0015
0.0020
Difference in mean proportions
2.597403e−02
2.597403e−02
1.515152e−02
p−value(wilcox test)
